# Lipid droplets in neurodegenerative diseases: pathological drivers and therapeutic vulnerabilities

**DOI:** 10.1038/s41420-026-03096-w

**Published:** 2026-04-09

**Authors:** Ourania Papapanagiotou, Kian Cotton, Christopher Edwards, David Michod, Lucy Crompton, Tim Craig, Maria Victoria Niklison-Chirou

**Affiliations:** 1 Life Science Department, University of Bath, Bath, UK; 2 Cancer Section, Development Biology and Cancer Programme, UCL Great Ormond Street Institute of Child Health, London, UK; 3 University of the West of England, Frenchay Campus, Coldharbour Lane, Bristol, UK; 4 Blizard Institute, Barts and The London School of Medicine and Dentistry, Queen Mary University of London, London, UK

**Keywords:** Multivesicular bodies, Endoplasmic reticulum

## Abstract

Lipid droplets (LDs) are dynamic intracellular organelles traditionally associated with energy storage, which have become increasingly recognised for their versatile roles in cellular metabolism and signalling. In the brain, LDs have emerged as critical regulators in neurodegenerative diseases (NDDs) such as Alzheimer’s disease (AD), Parkinson’s disease (PD), and Hereditary Spastic Paraplegia (HSP). LDs contribute to neurodegeneration by influencing lipid metabolism, oxidative stress, and inflammatory responses. For instance, in AD, dysregulated lipid metabolism and impaired Apolipoprotein E 4 (ApoE4) function lead to LD accumulation associated with neuroinflammation and amyloid plaque formation. In PD, interactions between LDs and α-synuclein suggest a potential link between lipid dysregulation and neurotoxicity. Mutations in LD-associated proteins, such as spastin and DDH2 in HSP, highlight the importance of proper LD regulation for neuronal health. While LD accumulation can be protective by mitigating lipotoxicity, prolonged dysregulation can exacerbate NDD pathology. Targeting LD metabolism, through enhancing lipophagy or modulating LD-associated proteins, represents a promising therapeutic avenue. This review highlights the dual roles of LDs in the brain, acting both neuroprotectively and neurotoxically, and the therapeutic potential of targeting LD dynamics for NDD treatment.

## Facts

- Are lipid droplets primarily protective or pathogenic in neurodegeneration—and what determines the switch?
- Are LD regulatory pathways (e.g., lipophagy, PLINs, seipin, DDHD2, spastin) viable and safe therapeutic targets in the human brain?
- Can LD accumulation serve as an early biomarker or stratification tool for neurodegenerative diseases?

## Introduction

Lipids are ubiquitous, water-insoluble molecules which fulfil numerous crucial physiological roles. These include key structural components of cell membranes, neutral energy stores for use in times of nutrient deprivation as well as intracellular and extracellular messaging as components of various hormones and vitamins [1]. Lipid droplets (LDs) are intracellular organelles that are expressed in almost all cell types [2]. Recently, they have garnered increased attention in relation to key lipid pathways such as storage, distribution and regulation of metabolism. Once considered solely inert storage units, it is increasingly clear that LDs are highly dynamic organelles with diverse functions [2]. While their roles in tissues like adipose and liver are relatively well-established, the full range of LD functions in various other tissues are still being unravelled.

In the brain, the second most lipid-rich tissue in the body [1], LDs have emerged as key players not only in homeostatic metabolic regulation of various cell types such as neurons, glial cells and astrocytes but also in the pathogenesis of diseases [2]. LDs are implicated in the development and progression of numerous neurodegenerative disorders (NDDs) such as Alzheimer’s disease (AD), Parkinson’s Disease (PD), Hereditary Spastic Paraplegia (HSP) and Amyotrophic Lateral Sclerosis (ALS). Their accumulation can trigger inflammatory responses and lead to neurotoxicity, contributing to disease progression [3]. Despite being at the centre of therapeutic research, until recently, patients with advanced-stage NDDs still receive therapeutic interventions targeting symptoms rather than the mechanisms underlying their pathology [4]. Whilst novel treatments such as Lecanemab and Donanemab have shown promise as successful therapeutics, their efficacy and safety remain questioned, opening the door for further research into novel treatments [5]. Given the potentially pivotal role of LDs in neurodegeneration, manipulating the biogenesis and metabolism of these organelles presents such an avenue for therapeutic interventions in NDDs. This review will explore the role of LDs in the pathogenesis of certain NDDs and the potential of targeting LD biodynamics as a therapeutic strategy.

## Lipid droplet composition

**Fig. 1:**
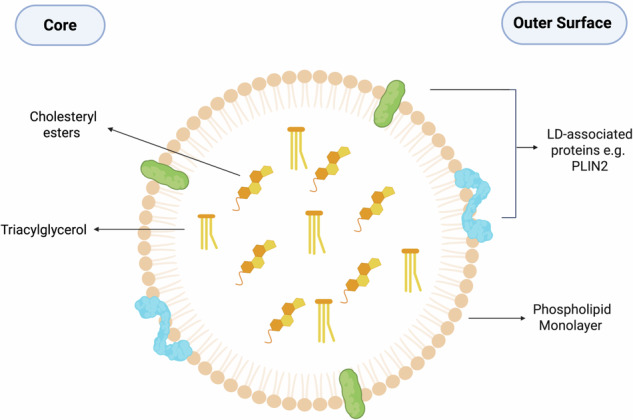
Lipid droplet composition. LDs have a core composed of neutral lipids, primarily triacylglycerol and sterol esters. This core is surrounded by a phospholipid monolayer. LD-associated proteins such as PLINs are embedded in this monolayer and play roles in LD dynamics, metabolism, and interactions with other organelles.

LDs are spherical organelles that vary in size, measuring from 100 nm to 100μm in diameter. Their core is composed of a variety of hydrophobic neutral lipids such as triacylglycerides (TAGs) and cholesteryl esters (CEs) [4] (Fig. 1). The outer surface of LDs comprises an amphipathic phospholipid monolayer [6], which can be enriched with various embedded LD-associated proteins, which are typically modified in the ER and are then targeted to LDs [7]. The main aim of these proteins is to maintain the structure, function, and morphology of LDs. Some of the most well-studied LD-associated proteins are perilipins (PLINs), part of the broader PAT-domain protein family, which are involved in LD dynamics and act by inhibiting lipases and hence LD breakdown [8]. In PLIN1 knockout mice, there is increased lipolysis and decreased lipid storage, highlighting the importance of these proteins in LD metabolism [9]. Both adipose triglyceride lipase (ATGL) and hormone-sensitive lipase (HSL), also known as cholesteryl ester hydrolase, are vital in lipolysis and the generation of free fatty acids (FFAs). Whilst these have generally been associated with adipocytes, their functions are now expanding in other tissues [10]. LD-associated proteins are essential for regulating the lifecycle of LDs, facilitating their interactions with other cellular structures, and maintaining energy homeostasis. Dysregulation or defects in these proteins can disrupt LD metabolism, leading to their accumulation and contributing to various pathologies.

## Lipid droplet metabolism

**Fig. 2:**
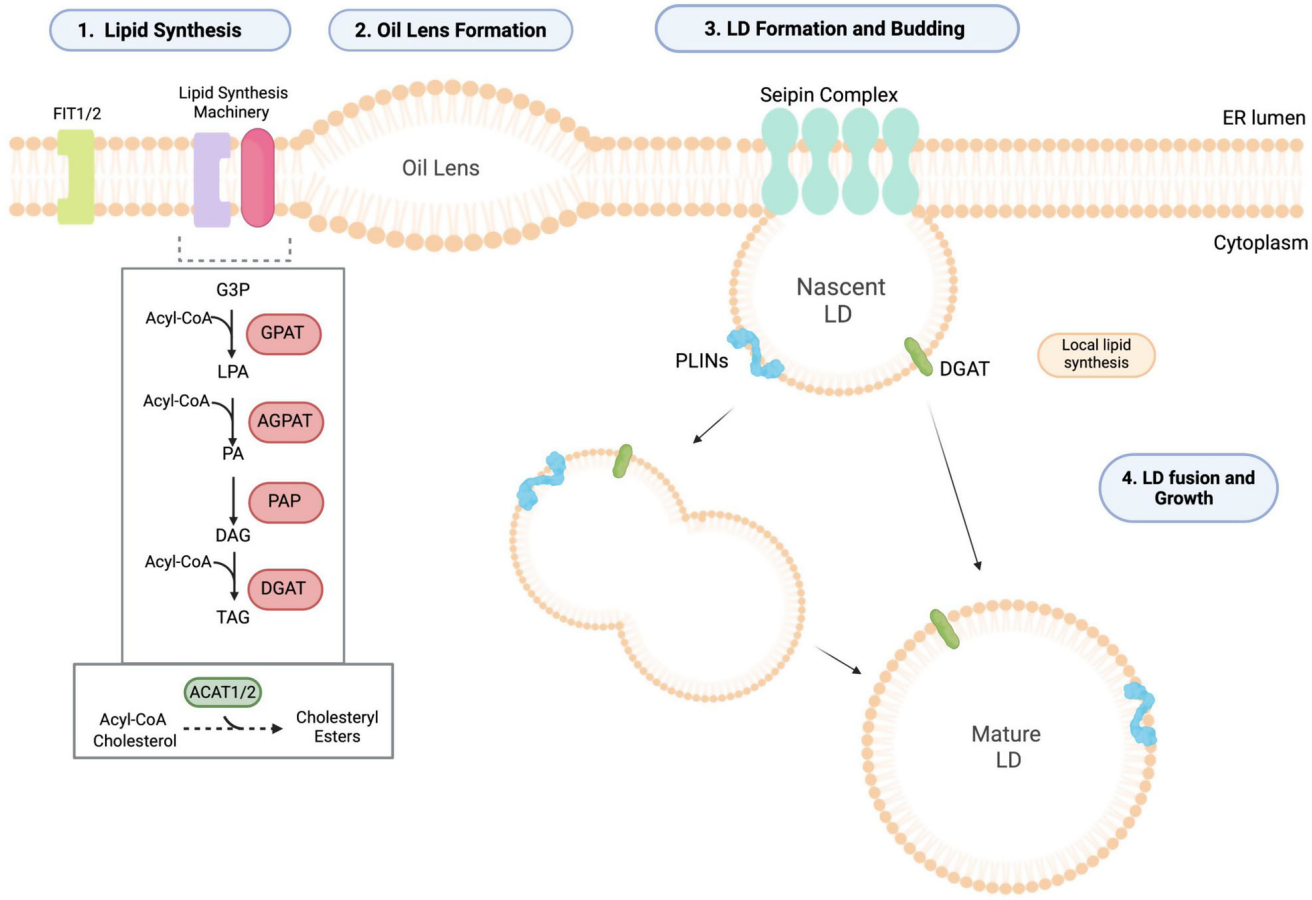
Lipid droplet biogenesis in the endoplasmic reticulum. Neutral Lipid synthesis and deposition between the outer and inner leaflet of the ER; inset illustrates Triacylglycerol (TAG) and sterol ester synthesis with the key intermediates and enzymes involved. FIT1/2 facilitate the formation of nascent LDs and the recruitment of enzymes essential for lipid synthesis. These proteins also ensure the correct maintenance of the composition and shape of the ER membrane. Seipin and other LD formation-associated factors are recruited to the oil lens, generated from the deposition of neutral lipids between the leaflets, leading to the formation of nascent LDs. LD budding and growth occur through fusion of multiple LDs or localised lipid synthesis, achieved by the action of certain LD-associated proteins, such as DGAT. Other LD-associated proteins include perilipins (PLINs), which play a vital role in LD metabolism. G3P Glycerol-3-phosphate, GPAT Glycerol-3-phosphate acetyltransferase, Acyl-CoA Acetyl Coenzyme A, LPA Lysophosphatidic acid, AGPAT Acylglycerolphosphate acyltransferase, PA Phosphatidic acid, PAP Phosphatidic acid phosphatase, DAG Diacylglycerol, DGAT Diacylglycerol acetyltransferase.

LD biogenesis occurs in the Endoplasmic Reticulum (ER) (Fig. 2) and is initiated by the synthesis of neutral lipids and oil lens formation, followed by the emergence of nascent LDs, cytoplasmic budding of the droplets, and growth. Once neutral lipids are synthesised through a series of enzymatic reactions, they are deposited in between the inner and outer leaflets of the ER. After reaching a certain concentration threshold, they form an oil lens. Nascent LDs are then formed, eventually budding off the ER [6].

Until recently, it was unknown whether oil lens formation and LD budding occurred at random or distinct regions of the ER. Recent findings in yeast have identified discrete LD-forming ER subdomains that support LD biogenesis, which are characterised by the presence of many different factors [11]. Seipin and LD assembly factor 1 (LDAF1), have been shown to create an LD assembly complex [12], assisting in the arrangement of these budding permissive sites. Seipin is especially crucial, as mutations in the *BSCL2* gene have been linked to conditions such as Spastic Paraplegia, wherein under certain mutations LDs have been found to be significantly enlarged [13, 14]. Its loss has also been coupled with supersized LDs [15]. Other proteins also have a vital role in LD formation, including Fat Storage Inducing Transmembrane protein 2 (FIT2), which recruits ER-tubule forming proteins such as REEP5 and Septins, contributing to membrane curvature and scaffold, essential for nascent LD formation. Furthermore, it facilitates the recruitment of lipid synthesis enzymes [6], and defects lead to LDs remaining embedded into the ER with no occurrence of budding [16]. After budding, LDs grow via the fusion of multiple LDs or localised lipid synthesis mediated by enzymes on their surface, such as DGAT [6]. While the formation of LDs is important, equally vital is their breakdown and lipid mobilisation, regulating lipid homeostasis within the cell, ensuring proper function and preventing pathological conditions.

LD catabolism is mainly executed by Adipose Triglyceride Lipase (ATGL), present on the surface of LDs, which hydrolyses TAGs into diacylglycerol and FFAs [17]. A secondary LD breakdown mechanism is lipophagy, the intracellular autophagic degradation of LDs. During lipophagy, LDs are sequestered by autophagosomes. This process can be facilitated by receptor-mediated recognition, for example, via the autophagy receptor p62/SQSTM1, which targets LDs for degradation by interacting with LC3 on the autophagosome membrane. Autophagosomes then fuse with lysosomes, leading to the breakdown of LDs by lysosomal acid lipases [18]. In starvation states the FFAs liberated during the breakdown of LDs are processed for energy supply via mitochondrial beta-oxidation, the TCA cycle, and oxidative phosphorylation [1]. Dysregulation of LD metabolism has been identified in many common NDDs where it precedes disease hallmarks, such as amyloid plaque formation in AD [19]. This highlights the importance of LDs in many tissues other than adipose, such as the brain, where their role is being researched more thoroughly.

## LDs in the brain and neurodegeneration

**Fig. 3:**
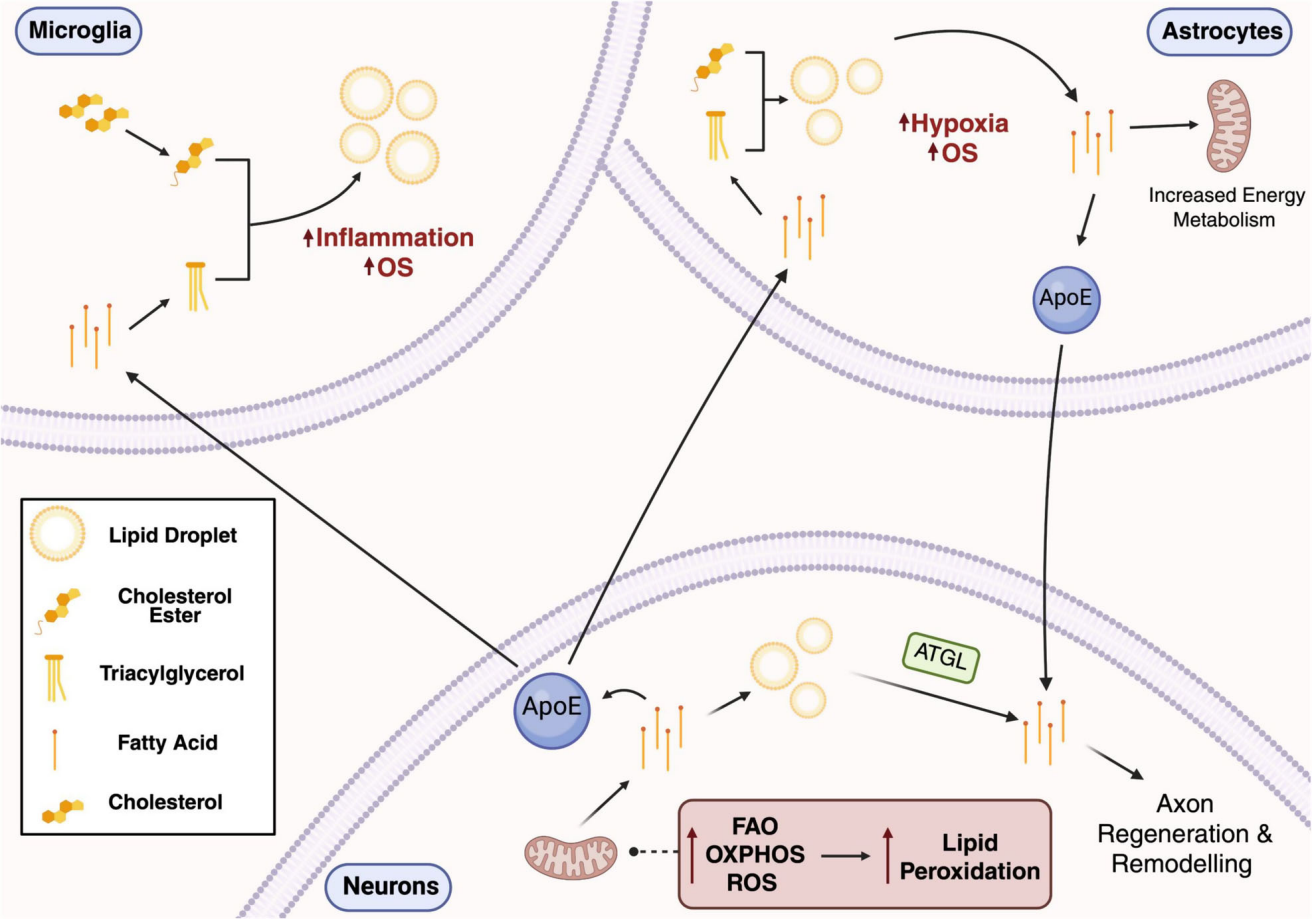
Cellular specificity of LD distribution and functions in the brain. Microglia, astrocytes, and neurons differentially regulate LD metabolism under cellular stress and continuously communicate to maintain lipid homeostasis. Microglia accumulate LDs, in part through ApoE-mediated lipid import from neurons, contributing to inflammatory responses and oxidative stress. Astrocytes loaded with neuronal fatty acids via ApoE form LDs under hypoxic or oxidative conditions and can release ApoE-associated lipids to neurons, supporting their energy metabolism as well as axon repair and remodelling. In neurons, fatty acid uptake enhances mitochondrial fatty acid oxidation and oxidative phosphorylation, but may also increase reactive oxygen species production and lipid peroxidation, which can be cytotoxic. To mitigate this lipid-induced toxicity, neurons export excess fatty acids via ApoE to astrocytes and microglia, where they are sequestered in LDs. ApoE apolipoprotein E, ROS reactive oxygen species, OXPHOS oxidative phosphorylation, FAO fatty acid oxidation, ATGL adipose triglyceride lipase. *Created with BioRender.com*.

LDs are present in a cell-specific manner in the brain (Fig. 3), predominantly found in glial cells, such as microglia and astrocytes [20]. Their presence increases in response to stress, ageing, or pathologies. For example, in microglia in the medial basal hypothalamus and ageing hippocampus, LD accumulation can be exacerbated by a high-fat diet and advanced ageing [21]. Furthermore, increased LD expression has recently been closely associated with the initiation and progression of Alzheimer’s disease (AD) and Parkinson’s disease (PD) [22, 23]. Indeed, Alois Alzheimer himself historically observed ‘oily spheres’ during postmortem analysis of his first patient.

LDs act as metabolic reservoirs by storing excess FFAs, which could lead to lipotoxicity. Their ability to sequester FFAs is particularly important for protecting cells from damage, especially neurons, as when in excess, FFAs can disrupt membrane integrity and be incorporated into toxic lipid entities, leading to apoptosis [24]. During periods of excitotoxicity, there is an increase in oxidative stress and the release of reactive oxygen species (ROS), which can lead to lipid peroxidation and neurotoxicity [25]. In this context, glial cells, particularly astrocytes, play a crucial neuroprotective role through a phenomenon known as neuron-glia metabolic coupling [26], during which glial cells essentially sequester FFAs and peroxidised lipids that have been received from neighbouring neurons to protect their viability. It has been shown that glial cells can increase the expression of genes, such as fatty acid-binding protein 7 (FABP7), neutralising the peroxidised FFAs generated by activated neurons. FABP7 binds the FFAs and sequesters them to mitochondria for β-oxidation or LDs for storage, hence reducing the overall oxidative stress state of neurons [27].

In microglia, the accumulation of LDs has been associated with impaired phagocytosis, increased ROS production, and the release of pro-inflammatory cytokines, leading to neuroinflammation [28]. Although the exact relationship between LDs and neuroinflammation is not fully established, studies suggest that LD accumulation in microglia may be both a cause and a consequence of inflammation. For instance, upon immune cell activation, LDs release stored lipids to fuel immune responses, including the synthesis of inflammatory mediators like eicosanoids and prostaglandins [29]. Furthermore, LD accumulation in microglia has been linked to the upregulation of enzymes involved in LD formation, such as DGAT2, leading to dysregulated inflammation and exacerbating neurodegeneration [30].

It is known that LDs play a protective role in the brain, primarily by preventing cellular damage. However, their accumulation beyond a certain threshold can become pathogenic, as observed in various NDDs and related mouse models. This dual nature of LDs highlights the importance of maintaining lipid homeostasis in the brain, and that dysregulation can be devastating for such pathologies.

## LDs in hereditary spastic paraplegia

**Fig. 4:**
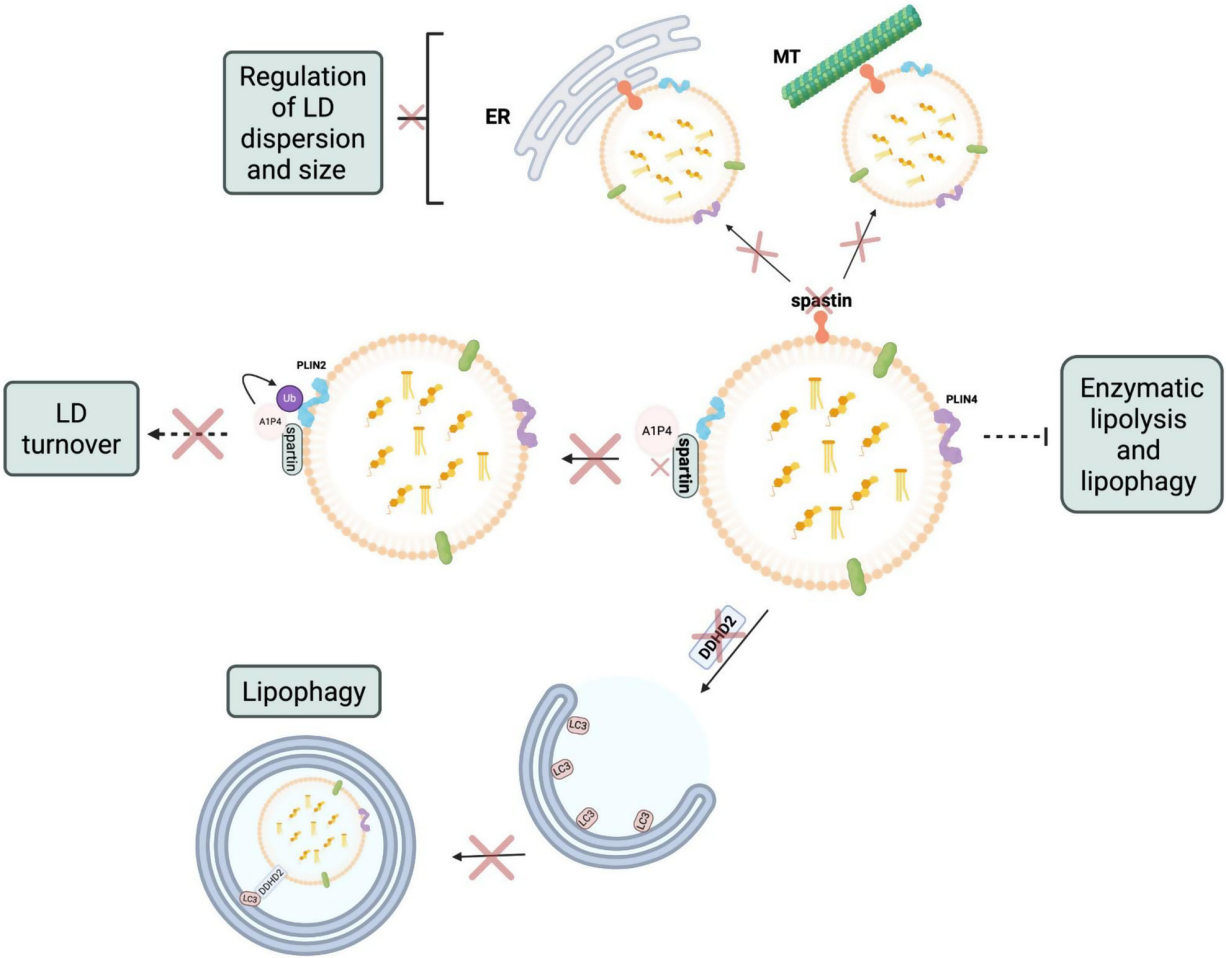
Lipid droplet (LD)-associated proteins dysregulated in neurodegenerative diseases (NDDs). Certain molecules involved in LD metabolism are often defective in some NDDs, leading to disturbed LD homeostasis. DDHD2 normally binds ATG8 family proteins, such as LC3, to promote lipophagy-mediated LD clearance. Spartin, another LD-associated protein, functions as an adaptor that recruits atrophin-1-interacting protein 4 (A1P4) to LDs, enabling ubiquitination of the LD coat protein PLIN2 and facilitating LD turnover. Mutations in DDHD2 and SPART impair these pathways, leading to defective LD clearance and LD accumulation. Perilipins (PLIN2 and PLIN4) coat LDs and protect them from enzymatic lipolysis and lipophagy; their upregulation, commonly observed in neurodegenerative diseases, further promotes LD persistence. Spastin acts as a molecular tether linking LDs to the endoplasmic reticulum (ER) and microtubules (MT). Disruption of these LD interactions leads to dysregulated LD dispersion and size. Red crosses indicate loss-of-function or inhibition. Created with BioRender.com.

Hereditary Spastic Paraplegia (HSP) is a group of inherited disorders marked by progressive leg weakness and stiffness, resulting from genetic mutations and degeneration of corticospinal axons [31]. Proteins such as spastin, DDH2 and spartin, linked to LD metabolism, are commonly mutated in HSP (Fig. 4).

The M1 isoform of spastin, a microtubule-severing protein, contains LD-targeting sequences that contribute to LD sorting in the ER and protein targeting to LDs, and mutations in this gene are seen in approximately 40% of HSP cases [32]. Papadopoulos et al. [33] found that spastin deficiency in *C. elegans* and Drosophila led to significant decreases in the number of LDs within the nerves, skeletal muscle and fat bodies of the Drosophila, as well as reduced TAG levels in the larvae. Therefore, suggesting that spastin, or the lack thereof, plays a key role in the regulation of lipid metabolism and LD biogenesis as a cause of this. Furthermore, defects in the M1 isoform of spastin have been associated with ER reorganisation, negatively impacting the dispersion of LDs within cells. In zebrafish treated with oleic acid, spastin loss resulted in excessive LD accumulation with reduced LD size [34]. These findings suggest that spastin defects lead to irregularities in LD dispersion, size, and number, which could contribute to HSP pathogenesis.

Another key factor in HSP pathogenesis linked to LDs is DDHD2, a triglyceride serine hydrolase (Fig. 4). Mutations in the *DDHD2* gene are a major cause of an HSP recessive complex form. DDHD2 has been shown to regulate LD dynamics by binding to ATG8 family proteins such as LC3 and promoting lipophagy. When this protein is defective, it leads to the accumulation of LDs [35]. Inloes et al. [36] demonstrated that DDH2 genetic knockout resulted in LD accumulation specifically in the central nervous system (CNS), but not in the periphery. This suggests that LD regulation may differ between the brain and other tissues, highlighting the importance of considering tissue-specific mechanisms when studying LDs in the context of NDDs.

Spartin, another HSP-associated protein, has been shown to interact with LDs. Mutations in the *SPART* gene contribute to the pathogenesis of Troyer Syndrome, a subtype of HSP. Normally, spartin functions as an adaptor protein that activates and recruits atrophin-1-interacting protein 4 (A1P4) to LDs. A1P4 ubiquitinates PLIN2, promoting LD turnover [37]. However, mutated spartin leads to LD accumulation in cultured human neurons and murine brain neurons [38], although the precise implications of this accumulation remain poorly characterised.

## LDs in Alzheimer’s disease

AD is the most prevalent NDD, it is primarily characterised by memory loss caused by the early death of hippocampal neurons, eventually spreading to other regions of the brain. AD is marked by the accumulation of extracellular amyloid-β plaques and intracellular hyperphosphorylated tau tangles [39]. The involvement of LDs in AD was first noted by Alois Alzheimer himself, observing adipose saccules in patients [40]. However, research into their role was limited until recent studies suggested significant links between LDs and amyloid plaque or tau formation and LD accumulation in AD brains and models, such as the work of Mi et al., [41] who observed accumulation of LDs following a breakdown in astrocytic FA degradation induced by knockout of *Tfam* in mice. Thus, leading to cognitive impairment through disruption of the astrocytic oxidative phosphorylation framework. LDs may also play a key role in neuroinflammation, now widely accepted as one of the pathological hallmarks of both AD and other NDDs: a recent study demonstrated that Aβ oligomers enhance lipid droplet formation and upregulated inflammatory phenotypes in astrocytes [42], thus directly linking AD pathological hallmarks, LDs and neuroinflammation.

Apolipoprotein E (ApoE) is a key protein involved in lipid transport, particularly cholesterol and TAGs, by binding to low-density lipoprotein (LDL) receptors and receptor-associated proteins, thereby facilitating the uptake of lipoprotein particles. In the brain, where neurons have limited capacity to break down FFAs, lipids are continuously shuttled to astrocytes via ApoE [43]. Among its allelic variants, ApoE4 is the most significant genetic risk factor for late-onset AD [44]. Compared to ApoE2 and ApoE3, ApoE4 has a reduced lipid-binding affinity and lower efficiency in lipid transport. Predominant ApoE4 presence in AD leads to dysregulated neuron-glial lipid transport and LD accumulation, potentially due to the reduced lipid-binding capacity [45]. Moreover, ApoE4-expressing microglia exhibit impaired mitochondrial oxidation and lipid metabolism, contributing to a pro-inflammatory state, exacerbating AD pathology [46]. Indeed, ApoE4/4 microglial have been demonstrated to accumulate LDs and display increased expression of the LD-associated enzyme ACSL1 [47]. Additionally, conditioned media from ACSL1-positive microglia induces tau phosphorylation in neuronal cells, implying a critical role in AD pathology.

Evidence of LDs in neurones themselves is more limited; however, some recent studies have demonstrated that ApoE4 neurons accumulate LDs, which in turn elevate cholesterol levels and phosphorylated tau (p-tau) [48]. Additionally, direct observation of LD dynamics from hiPSC-derived neurones has demonstrated that tau protein pathology amplifies LD accumulation, with unsaturated lipids transferred from neurons to microglia. In this study, neuronal LD accumulation was driven by tau pathology, inhibiting AMPK, an inhibitor of LD synthesis, thus increasing lipid synthesis and LD formation [49].

Another genetic link between LD metabolism and AD is the loss-of-function mutation (*R47*) in the triggering lipid receptor expressed by microglial cells, TREM2. Normally, TREM2-activated microglia phagocytose the amyloid plaques, alleviating AD pathology and regulating lipid uptake [50]. In *R47* seen in AD, the normal function is compromised, leading to LD accumulation, diminished plaque proximity and reactivity, leading to the disease progression [51].

These findings suggest that targeting LD dynamics, lipid transport and neuroinflammation may offer promising therapeutic approaches for AD by restoring neuron and glial lipid homeostasis, enhancing microglial clearance of pathological aggregates, and reducing the metabolic stress and oxidative damage associated with LD accumulation—ultimately interrupting the vicious cycle linking lipid dysregulation to amyloid deposition, tau pathology, and neurodegeneration.

## LDs in Parkinson’s disease

PD, the second most prevalent NDD after AD, manifests in symptoms such as rigidity, tremors and cognitive impairment. This is the result of the progressive degeneration of dopaminergic neurons in the substantia nigra, heavily impacted by the intracellular aggregation of a-synuclein, leading to the formation of Lewy Bodies [52]. For years, PD has been thought to be a proteinopathy due to research heavily focusing on a-synuclein aggregates. However, new research focusing on the lipid aspect of Lewy bodies, making links between PD and LD metabolism dysregulation, has led to PD being characterised as a potential lipidopathy [53]. Indeed, many different genes involved in lipid metabolism have been identified as risk factors for PD, including GBA1 and SMPD1 [54].

Under physiological conditions, a-synuclein interacts with membranes, whose composition impacts its tendency to aggregate [55]. One such membrane is the LD phospholipid monolayer. α-synuclein is involved in LD metabolism, protecting them from lipolysis, hence causing their accumulation [56]. Interestingly, this is executed less efficiently by the PD-associated mutants, A30P and A53T [57]. Initially, LD accumulation might act protectively, alleviating excess oxidative stress by sequestering FFAs, supporting mitochondrial function and cellular vitality [2]. Eventually, LD overload leads to the formation of insoluble a-synuclein aggregates, resistant to proteolytic digestion [58, 59]. Disrupted lipid homeostasis and excessive a-synuclein aggregation induce death of dopaminergic neurons, especially susceptible to metabolic damage [60]. Of note, neurons and microglia in PD have been shown to exhibit abundant LD accumulation [61]. Some studies report low LD levels in astrocytes alongside high LD levels in neurons in PD, suggesting impaired metabolic coupling between these cell types. In contrast, other studies have found LD buildup in astrocytes, which may interfere with their ability to neutralise aminoacyl-induced neurotoxicity in dopaminergic neurons [62]. Another implicating factor is PLIN4, which safeguards LDs from lipolysis and turnover. Han et al. [63] showed that PLIN4 was upregulated in PD models, leading to LD formation and progression of PD.

Conversely, other studies have suggested a potentially intriguing protective effect of LDs in PD. Alarcon-Gil et al. [64] indicated that linoleic acid combats PD by stimulating LD formation and accumulation. While this conflicting data suggests a robust link between LD accumulation, metabolism, a-synuclein and PD, further investigation is needed to determine whether underlying mechanisms progress PD pathogenesis or act protectively. Exploring therapeutic interventions targeting LDs and their metabolism could aid this investigation.

## The therapeutic potential of LDs

Despite significant research efforts being directed towards the development of treatments for NDDs, they remain incurable and devastating [65], with clinical characteristics heavily impacting patients’ lives and imposing a huge healthcare and familial burden. Most therapeutics for NDDs, such as Levodopa for PD, ameliorate symptoms, rather than targeting the pathological cause of the disease [66]. Bearing in mind the heavily implicated roles of LDs in NDDs, it would be fair to assume that they could present a valid therapeutic target and warrant further investigation. Recently, Yang et al. [67] explored the potential of a marine-derived natural polyketide, tetrahydroauroglaucin, which showed neuroprotective potential by decreasing LD accumulation through upregulating the RUBCN autophagy pathway. Another strategy was employed by Han et al. [68], who used small-molecule flavonoid Kaempferol on a PD mice model, reducing LD accumulation by activating lipophagy. Tang et al. [69] observed that overexpression of neuroprotective protein parkin led to the activation of a major regulator of FA and sterol synthesis, SREBP2, which upregulated lipoprotein lipase and promoted lipolysis. Current studies show very promising results, with some showing improved NDD clinical manifestation, suggesting that targeting LDs could be beneficial. However, as with most conditions of such complexity, it is likely that any treatment targeting LDs metabolism would need to be utilised synergistically alongside current treatments.

## Conclusions and future directions

The scientific community has increasingly recognised the importance of LDs in the brain, where they participate in diverse physiological processes and play significant roles in the pathogenesis of many NDDs. While LDs can serve protective functions such as storing excess FFAs and mitigating lipotoxicity, their dysregulation and accumulation can contribute to the development and progression of NDDs like HSP, AD, and PD. Given the pivotal involvement of LDs in these pathologies, there could be great potential in targeting LD metabolism as a novel therapeutic strategy, already explored to some extent, with promising findings. However, the complex pathogenesis of NDDs necessitates further investigation into precise mechanisms and the potential benefits of combination therapeutic regimens. In the future, the potential use of LDs as biomarkers could also be investigated outside of therapeutic targeting, especially in contexts like AD where LD accumulation can precede other disease hallmarks. Understanding the delicate balance of LD metabolism in the brain will be crucial for developing effective treatments for these debilitating conditions.
